# Dual role of Endoplasmic Reticulum Stress-Mediated Unfolded Protein Response Signaling Pathway in Carcinogenesis

**DOI:** 10.3390/ijms20184354

**Published:** 2019-09-05

**Authors:** Natalia Siwecka, Wioletta Rozpędek, Dariusz Pytel, Adam Wawrzynkiewicz, Adam Dziki, Łukasz Dziki, J. Alan Diehl, Ireneusz Majsterek

**Affiliations:** 1 Department of Clinical Chemistry and Biochemistry, Medical University of Lodz, 90-419 Lodz, Poland (N.S.) (W.R.) (A.W.); 2 Department of Biochemistry and Molecular Biology, Hollings Cancer Center, Medical University of South Carolina, Charleston, SC 29425, USA (D.P.) (J.A.D.); 3 Department of General and Colorectal Surgery, Medical University of Lodz, 90-419 Lodz, Poland (A.D.) (Ł.D.)

**Keywords:** cancer, carcinogenesis, endoplasmic reticulum stress, protein kinase R (PKR)-like endoplasmic reticulum kinase (PERK), unfolded protein response, apoptosis, reactive oxygen species, cancer treatment

## Abstract

Cancer constitutes a grave problem nowadays in view of the fact that it has become one of the main causes of death worldwide. Poor clinical prognosis is presumably due to cancer cells metabolism as tumor microenvironment is affected by oxidative stress. This event triggers adequate cellular response and thereby creates appropriate conditions for further cancer progression. Endoplasmic reticulum (ER) stress occurs when the balance between an ability of the ER to fold and transfer proteins and the degradation of the misfolded ones become distorted. Since ER is an organelle relatively sensitive to oxidative damage, aforementioned conditions swiftly cause the activation of the unfolded protein response (UPR) signaling pathway. The output of the UPR, depending on numerous factors, may vary and switch between the pro-survival and the pro-apoptotic branch, and hence it displays opposing effects in deciding the fate of the cancer cell. The role of UPR-related proteins in tumorigenesis, such as binding the immunoglobulin protein (BiP) and inositol-requiring enzyme-1α (IRE1α), activating transcription factor 6 (ATF6) or the protein kinase R (PKR)-like endoplasmic reticulum kinase (PERK), has already been specifically described so far. Nevertheless, due to the paradoxical outcomes of the UPR activation as well as gaps in current knowledge, it still needs to be further investigated. Herein we would like to elicit the actual link between neoplastic diseases and the UPR signaling pathway, considering its major branches and discussing its potential use in the development of a novel, anti-cancer, targeted therapy.

## 1. Introduction

In this day and age, cancer and other types of neoplastic growths have become a global health problem, since many of them bear hallmarks of rapid progression and poor prognosis [1,2]. According to the World Health Organization (WHO), the cancer burden is estimated to have risen in 2018 with about 18.1 million new cases of cancer diagnosed globally. It has been also reported that 9.6 million people worldwide died from cancer in 2018, which made it the second leading cause of death. In other words, approximately 1 in 6 deaths is due to cancer, whereas around one third of them are due to the leading dietary and behavioral risk factors such as alcohol and tobacco abuse, overweight, low fruit and vegetable intake, and physical inactivity.

Despite surgical operation being the main effective method of medical treatment for solid tumors, such as colorectal cancer (CRC), adjunctive therapies including chemotherapy and radiotherapy still remain essential for cancer patients, especially those suffering from blood cancer or for patients in advanced stages of disease [3,4,5,6,7]. Unfortunately, chemotherapy is currently considered imperfect and non-specific, associated with long-term, irreversible side-effects [8,9,10,11], which in the worst-case scenario may increase the risk of development of another types of cancer later on in patients’ life [12]. For instance, Paclitaxel (PTX), a chemotherapeutic agent commonly known and administered over the years for treatment of lung, pancreatic, breast, ovarian and many other cancer types [13,14], has been reported as causing hypersensitivity reactions, myelosuppression, peripheral neuropathy, or even infertility due to the ovarian damage [12,15]. In addition, its low solubility makes it difficult to applicate in vivo. Another antineoplastic, alkylating agent, Cyclophosphamide (CPA), mostly used along with cancer treatment in bone marrow transplantation and certain autoimmune disorders, has proved to be highly toxic not only for the targeted cancer cells, but also for normal cells of the human body [16,17]. Moreover, the mentioned drug acts especially on the myocardium and endothelial cells, which leads to cardiomyopathy, myocardial infarction and even heart failure, thus demonstrating cardiotoxic properties [17]. Interestingly, a study by Bhat et al. (2018) revealed the harmful effects of CPA on liver and kidney tissues, depending on duration of treatment and progressive increase in dosage. Therefore, it becomes vital to monitor liver and kidney functions in patients undergoing a chemotherapeutic regimen [16]. Moreover, exposure to radiation is a well-documented risk factor in the development of tumors such as papillary thyroid cancer, considering the fact that ionizing radiation is likely to induce significant DNA damage, which results in new mutations [18]. There is also a great interest in Genistein (Gen), given its antioxidant, antiradical and antiproliferative activity [19], which is particularly effective against hormone-dependent types of cancer [20]. However, its instability under various conditions such as high temperature, pH, presence of oxygen, as well as low solubility in water, oral bioaccessibility and bioavailability hinder potential clinical effectiveness [21,22].

Therefore, further research in the field of signaling pathways to discover novel, anti-cancer drugs and thereby improve currently used treatment strategies and minimize their side-effects, has clearly become necessary.

## 2. UPR in Cancer Initiation and Progression

Endoplasmic Reticulum (ER) is commonly known as an organelle responsible for regulation of protein, lipid, and steroid synthesis and calcium-dependent signaling [23,24,25]. Adequate oxygen supply is required to permit disulfide bonds formation via cysteine oxidation. It is essential for the proper conformation of the majority of proteins [26]. The quality control systems of the ER selectively regulate trafficking of those proteins which are correctly-folded and target the misfolded ones for proteolysis simultaneously. However, under certain circumstances, the protein degradation process is insufficient, which results in accumulation of the misfolded and unfolded proteins within the lumen of the ER [27]. Numerous conditions, including nutrient deprivation, hypoxia, acidosis, drug-induced toxicity and irradiation, may interfere with protein folding, contributing to unfolded or misfolded protein accumulation and, in consequence, ER stress development [28,29,30]. At that point, the proteins can still achieve correct conformation through further processing by ER chaperones, such as calreticulin, calnexin, and ER resident protein 57 (ERp57). Otherwise, they are sent to so-called ER-associated degradation (ERAD) by means of ubiquitin-dependent proteolytic degradation, or undergo autophagy [23]. As a response to prolonged ER stress conditions, the Unfolded Protein Response (UPR) signal transduction cascade is directly activated to counteract the occurring damage. The UPR is itself mainly cytoprotective, aiming to alleviate the damage and restore cellular homeostasis via transcriptional induction of specific molecular chaperones [31]. Other than that, mentioned UPR response plays a major role in regulation of the expression of genes responsible for calcium and redox homeostasis, protein trafficking, ER quality control, autophagy, and lipid synthesis [27]. Interestingly, the UPR signaling pathway may switch to death-triggering pathways and lead to apoptosis through the protein kinase R (PKR)-like endoplasmic reticulum kinase/eukaryotic translation initiation factor 2α/activating transcription factor 4 (PERK/eIF2α/ATF4) axis and subsequent activation of C/EBP homologous protein transcription factor (CHOP) [28]. Above-mentioned event may occur in the case of prolonged activation of PERK-dependent UPR signaling pathway under extensive and severe ER stress conditions [29]. Thus, the life-versus-death determination or coordination of adaptive and apoptotic responses are tightly regulated.

Cancerous cells remain highly proliferative despite their inadequate vascularization. They demonstrate high metabolism necessary to support malignant expansion and create a specific microenvironmental niche consisting of low pH, hypoxia, and restricted nutrients supply [23]. In order to adapt to low glucose level, tumor cells switch to a high rate of aerobic glycolysis also known as the Warburg effect [32]. This results in lactic acidosis and subsequent pH reduction, which aggravate local distress. The above-mentioned conditions are crucial to promote cancer survival and progression by means of the downregulation of CHOP and several B-cell leukemia/lymphoma-2 (BCL-2) family members [27]. On the contrary, a nutrient-poor environment constitutes the main obstacle cancer cells have to face in order to survive, having already developed mutations to overcome cell cycle and apoptotic checkpoints [28].

The other key factor that makes the tumor surrounding widely cytotoxic is the excessive intracellular generation of Reactive Oxygen Species (ROS) within the mitochondria, which could be also be induced by enhanced activation of the phosphoinositide-3 kinase/protein kinase B (PI3K/Akt) signaling pathway. ROS have been shown to impair disulfide bonds formation, protein glycosylation as well as ATP production, directly leading to the accumulation of misfolded and unfolded proteins within the Endoplasmic Reticulum (ER) lumen and subsequent activation of the Unfolded Protein Response (UPR) signaling pathway [23]. In normal cells, the above-mentioned signaling pathway may be triggered by numerous stressors such as irradiation, deficiency of energy, increased levels of Ca^2+^ or ROS.

The first known mechanism of UPR signaling pathway activation involves ER oxidase 1α (ERO1α), an enzyme which is induced under hypoxic conditions to support protein folding via catalyzing oxygen-dependent disulfide bond formation. Another UPR-activating mechanism is the glycogen synthase kinase 3B (GSK3B) up-regulation, which in turn contributes to the activation of the PERK-dependent branch of the UPR signaling pathway [33]. As a consequence, cancer cells certainly share higher expression of PERK and its main downstream effectors including phosphorylated eukaryotic translation initiation factor 2α (p-eIF2α), ATF4, CHOP and nuclear factor-like 2 (Nrf2) [29,34]. One of them, ATF4, promotes tumor cells proliferation via limiting oxidative DNA damage.

It has been proven that hyperphosphorylation of PERK is crucial for cell survival under extreme hypoxia as well as the enhanced expression of wild-type PERK increases the eIF2α phosphorylation level. Experimental inactivation of PERK in cells have resulted in decreased hypoxia-induced phosphorylation of eIF2α and have reduced translational attenuation compared to wild-type cells [35].

In general, PERK major effectors contribute to ER stress-mediated death of normal cells under standard conditions. However, cancer cells have evolved a way to adapt to prolonged ER stress due to pro-survival alterations in the UPR signaling pathway. The above-mentioned property gives them the ability to avoid apoptotic cell death as well as acquire a chemoresistant phenotype [23]. In other words, the UPR signaling pathway activated within cancer cells can be described as both sustained and non-lethal [36]. The specific microenvironment might also be enhanced by the release of the pro-inflammatory mediators from tumor cells. It results in the recruitment of immune cells which are involved in angiogenesis, invasion, as well as metastasis [37]. It has been proven that this phenomenon, called transmissible ER stress (TERS), might be influenced by the sustained activity of inositol-requiring enzyme-1a (IRE1α), which represents one of the main sensors of the UPR signaling pathway [38]. Hence, the UPR signaling pathway is said to regulate the balance among cancer cell death, dormancy promotion, aggressive growth, as well as impinge on the sensitivity to chemotherapeutics or host protection by inducing apoptosis. Due to the UPR signaling pathway, the ER protein-folding capacity and proteostasis are maintained during tumorigenesis. Simultaneously, apoptosis is counteracted and, under favorable conditions, recurrence is permitted [39]. Neoplastic cell apoptosis ensues only when ER stress is severe or prolonged and attempt to restore disrupted ER proteostasis [27].

**Figure 1:**
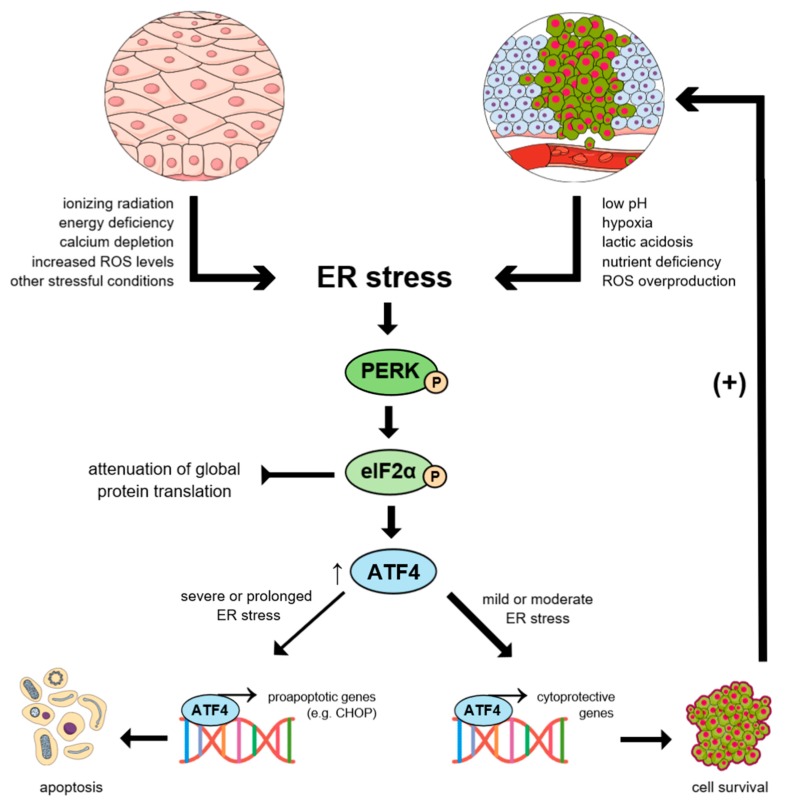
Dual response to Endoplasmic Reticulum (ER) stress conditions in normal epithelial cells and cancer cells via the protein kinase R (PKR)-like endoplasmic reticulum kinase (PERK)-dependent Unfolded Protein Response (UPR) signaling pathway activation. Numerous stress-inducing factors may contribute to impaired formation of proteins and their subsequent accumulation, resulting in activation of UPR signaling pathway in both cell types. While in normal cells their origin is mainly extracellular and rather occasional, cancer cells are shown to be permanently exposed to self-induced, chronic stressors as an effect of their own metabolism. This is the reason why they developed adaptive mechanisms to deal with severe stress conditions and avoid apoptosis, which results in a positive feedback loop. The PERK/eukaryotic translation initiation factor 2α/activating transcription factor 4 (PERK-eIF2α-ATF4) axis is certainly involved in both pro-survival and pro-apoptotic signaling pathways.

In addition, the UPR activation is not only involved in carcinogenesis, but also in a wide range of other human diseases, such as Alzheimer’s, Parkinson’s, prion-related diseases, type II diabetes, atherosclerosis, and glomerulopathies, directly leading to significant cell degeneration and organ damage [40,41,42,43]. For all these reason, further, detailed investigation is required in order to clarify which stages of tumor development UPR regulates exactly, how its modulation alters the balance between cell death and survival, and to finally determine how the fate of a tumor cell is eventually decided [28] (Figure 1).

## 3. Molecular Mechanism of the UPR Signaling Pathway Activation

### 3.1. BiPs as the Major Regulators of the UPR Signaling Pathway

PERK, transmembrane activating transcription factor 6 (ATF6) and IRE1α constitute a transmembrane receptors, forming inactive, stable complexes with the binding immunoglobulin proteins (BiP)/78 kDa glucose-regulated protein (GRP78) [44,45]. The mentioned proteins play a key role in the activation of three central branches of the UPR signaling pathway. Interestingly, PERK is considered the major regulator of the UPR signaling pathway, which determines cell fate, indicating whether the cell will undergo apoptosis or not [46]. BiP chaperones generally serve as foldases which prevent misfolded proteins from being further transported or aggregated, resulting in UPR activation [27]. When misfolded proteins bind to BiP chaperones, their subsequent dissociation from the complex occurs, resulting in the phosphorylation of the three transducers and the activation of their respective UPR signaling pathways. At this stage, the complex is still capable of reversing to its inactive state independently from any external phosphatase involvement. Each of the branches possesses its unique downstream targets leading to an appropriate response on the molecular level, ranging from adaptive to maladaptive. The decisive molecular mechanism for switching between these two different responses, the alterations in responses to various stress conditions, and the cross-talk between these three branches have yet to be uncovered [47].

Beyond the enhanced expression of the GRP78 proteins in the ER, they can be expressed either in other organelles or on the cell surface (sGRP78), where it may regulate the transforming growth factor-β (TGFβ) and PI3K signaling pathway [48,49,50]. It is worth mentioning that BiP chaperones can act both as a positive and negative regulator of the UPR, which indicates their prominent role in the development of cellular adaptation to ER stress. According to the mathematical model provided by Erguler et al. (2013), adaptation is conducted only until the limit of BiP synthesis is reached. Under severe or prolonged ER stress conditions, BiP fails to suppress UPR activation, so that the BAX/BAK/BH3 (respectively: BCL-2-associated X protein, Bcl-2 homologous antagonist/killer, and Bcl-2 homology domain 3) apoptotic signals become, in turn, irreversibly activated [47].

### 3.2. Dual Function of the IRE1α Receptor in Cell Fate Determination

There are two commonly known isoforms of IRE1, IRE1α, and IRE1β, whereas the first one is expressed ubiquitously and the second one is tissue restricted [51,52]. Therefore, IRE1α emerges as more significant in current research and is generally defined as a positive regulator for cell survival. Accordingly, during irremediable ER stress, its signaling becomes terminated in order to enable apoptosis [51,53]. Thus, IRE1α is responsible for promotion of either cell survival or apoptotic cell death [54,55].

IRE1α, which is an ER transmembrane protein, serves as a specific stress sensor, which monitors proteostasis through N-terminal, ER luminal domain bound to BiP, and triggers UPR signaling pathway activation through C-terminal, cytoplasmic kinase, and RNase domain [56]. Physical interaction with BiP represses IRE1α when ER homeostasis is maintained. Upon ER stress, IRE1α monomers oligomerize, triggering sequential autophosphorylation, conformational change, higher order assembly and finally activation of the RNase domain [57]. Its subsequent interaction with TNF receptor-associated factor 2 (TRAF2) leads to activation of the major pro-apoptotic mediators, such as c-Jun N-terminal kinase (JNK), the key activators of pro-apoptotic BCL-2 family members (inter alia, the BH3 interacting-domain death agonist, BID, or Bcl-2-associated death promoter, BAD), as well as the caspase-12 protein [45,58]. The RNase domain can initiate two diverse downstream UPR signaling pathways: Through the unconventional splicing of *X-box binding protein 1 (XBP1)* mRNA or posttranscriptional modifications of manifold substrates promoted by regulated IRE1α-dependent decay (RIDD), respectively [55,56].

XBP1 spliced by IRE1α (XBP1s) enters the nucleus to induce transcription of the UPR target genes and, in turn, triggers adaptive reactions, including, inter alia, upregulation of ER chaperones and ERAD ubiquitination machinery [59]. Moreover, activated XBP1s dimerizes with the hypoxia-inducible factor 1α (HIF1α) to potentiate the expression of hypoxia-responsive genes including *vascular endothelial growth factor A* (*VEGF-A*), a key mediator of angiogenesis [60]. XBP1s also directly interacts with *Myc* proto-oncogene, which either drives IRE1α expression and XBP1 splicing, or potentiates XBP1s transcriptional activity [61]. Furthermore, XBP1s may enhance catalase expression and its loss adequately sensitizes cells to stress-induced, oxidative apoptosis. Above-mentioned event takes place due to the association of catalase deficiency in cells with ROS generation and p38 activation [62].

Under irremediable ER stress conditions, the splicing of XBP1 mRNA ceases and instead, IRE1α conducts selective cleavage and thus the degradation of mRNAs encoding ER-related proteins [55,63,64]. This phenomenon called RIDD appears to be essential to promote cell survival via limiting the number of redundant peptides entering *the* ER [45]. However, once the ER stress intensifies, RIDD may promote cell death via enhancing degradation of pro-survival protein encoding mRNAs [45,64], the main one commonly known as *anti-Casp2* [29]. As a result, the activation of apoptotic initiator caspase-2 follows, directly leading to mitochondrion-dependent apoptosis [63].

Interestingly, recent studies have shown that both ATF6 and PERK-ATF4 signaling axes contribute to increased XBP1s mRNA expression via the activation of the IRE1α-XBP1 pathway in two separated mechanisms. This interplay may enable cells to adapt to various types and levels of stress through the modulation of the IRE1α-XBP1 pathway [65]. Conversely, it has been proven that IRE1α deficiency unexpectedly causes a decrease in the expression of eIF2α through PERK-dependent autophagy, resulting in increased cell death [66]. Another finding has demonstrated that IRE1α signaling has an ATF6-dependent off-switch, since loss of ATF6 results in uncontrolled IRE1α activity with increased XBP1 splicing during ER stress [67].

### 3.3. The Role of the ATF6 in Proteostasis Restoration

Similar to IRE1α and PERK, ATF6 contains a stress-sensing, ER luminal domain as well as an enzymatic, cytosolic domain [51]. It has been also confirmed that ATF6 exists in two isoforms, ATF6α and ATF6β [52]. Upon ER stress activation, ATF6 is released from BiP and relocated to the Golgi apparatus, where it is subsequently cleaved by site-1 and site-2 proteases (S1P; S2P). Thus, the cytosolic domain of ATF6, which is a transcription activator for XBP1, BiP, CHOP and other chaperones, becomes activated in order to promote protein-folding homeostasis [68,69,70]. Next, the cleaved transcription factor domain of ATF6 (ATF6f) enters the nucleus in order to modulate transcription of the UPR target genes [51]. ATF6- and IRE1α-mediated branches of the UPR signaling pathway are interconnected, since both of them upregulate either XBP1, involved in BiP synthesis, protein folding and quality control, or ERAD-associated proteins [23]. ATF6 is believed to mainly induce cytoprotective response, comprising ER biogenesis, expression of chaperones and protein degradation, although there was found a link between it and the indirect downregulation of a pro-survival BCL-2 family member, myeloid cell leukemia sequence 1 (MCL1) [71]. Additionally, the modulation of ATF6 is sensitively tuned so that it adjusts the ER capacity to match demand without globally influencing protein processing [72].

### 3.4. Molecular Mechanism of the PERK-mediated Activation of the UPR Signaling Pathway

Once ER stress is triggered, a dissociation of the BiPs from PERK takes place, ultimately resulting in its dimerization, autophosphorylation, and activation. Subsequently, PERK-dependent phosphorylation of eIF2α follows, which results in a transient block of global mRNA translation to decrease the protein-folding demand in the ER [73,74]. Phosphorylated elF2α also evokes arrest of the cell cycle via the accelerated depletion of cyclin D1 and selectively enhances the translation of specific mRNAs including ATF4, ATF5, ATF6, XBP1, CHOP, and cellular inhibitor of apoptosis protein 1 and 2 (cIAP1/2) at the same time [68,75,76,77].

ATF4, apart from being the major downstream target of PERK-dependent phosphorylation of eIF2α, constitutes a member of the cAMP response element-binding protein CREB/ATF family, ubiquitously expressed in cells [78]. ATF4 plays a key role in transcriptional regulation of the pro-survival genes, related mainly to oxidative stress, autophagy, protein folding, amino acid synthesis, and cell differentiation [23,79]. Thus, ATF4 constitutes a crucial mediator of both oxidative and metabolic homeostasis, which predominantly promotes cell survival [80,81]. ATF4 is also responsible for regulation of *tribbles homolog 3 (TRIB3)* [82], *unc-51-like autophagy activating kinase 1 (ULK1)* [83], and *lysosomal-associated membrane protein 3 (LAMP3)* [84], which constitute genes mobilized in hypoxic conditions via the PERK-ATF4 signaling pathway [68]. In various microenvironmental stress conditions, for instance, hypoxia, toxic exposure, starvation or nutrients deprivation, the ATF4 level becomes significantly elevated [78].

Other proteins upregulated by p-eIF2α, such as VEGF-A (important to compensate chronic hypoxia) or the growth arrest and DNA damage-inducible protein, GADD34 (which dephosphorylates p-eIF2α in negative feedback loop) are regarded as essential in overcoming cell stress [85]. The latter is directly transactivated by one of the ATF4 targets and downstream PERK effectors, CHOP, resulting in growth arrest and global protein synthesis restoration. Besides, CHOP promotes cell death via direct upregulation of pro-apoptotic proteins when ER stress conditions are severe or prolonged, which may be caused by the excessive ROS formation [27].

It has been reported that the activation of the PERK-dependent UPR signaling pathway may be confirmed by the elevation of GRP78, p-PERK, p-eIF2α, and CHOP expression [86]. Thus, the transcription of numerous genes encoding ER chaperones and enzymes critical to repair the stress-caused damage and cope with the excessive amount of unfolded proteins is selectively induced. Otherwise, the apoptotic cell death occurs, with a reduced overall transcription rate [85].

### 3.5. Nrf2 is Engaged in Multiple Antioxidant Responses

The Nrf2 constitutes a transcription factor and member of Basic Leucine Zipper Domain (bZiP) proteins. The study by Cullinan et al. (2003) has demonstrated that Nrf2 is directly phosphorylated in the PERK-dependent manner [87]. There is ample evidence that Nrf2 upregulates the expression of antioxidant proteins, primarily enzymes involved in biosynthesis and metabolism of the most relevant cellular antioxidant, glutathione (GSH): glutamate cysteine ligase (GCL), glutathione synthetase (GSS), glutathione peroxidase 2 (GPx2) and glutathione S-transferases (GSTs) [88]. It has also been reported that Nrf2 promotes the expression of *specific cystine/glutamate antiporter* (*Xc-*), which enhances the import of GSH substrate cysteine [89] and enzymes involved in detoxification processes, such as *heme oxygenase-1* (*HO-1*) [90] and *NAD(P)H quinone oxidoreductase 1* (*NQO1*) [91].

Nrf2 is also responsible for enhanced expression of ATP-binding cassette (ABC) transporters, including multidrug resistance-associated protein (MRP) family members, which determine efflux of ions, toxins and signal transduction [92], and xenobiotic-activated receptor (XAR), capable of activating the antioxidant response element pathway (ARE) [93]. In addition, a recent study has investigated a potential interconnection between the Nrf2-ARE axis and nuclear factor kappa-light-chain-enhancer of activated B cells (NF-ĸB), a nuclear factor which regulates cytokine production and cell survival, in the context of neuroinflammatory and neurodegenerative disorders [94].

A component of ubiquitin ligase complex, Kelch-like ECH-associated protein 1 (Keap1), represents a negative regulator of the Nrf2 under quiescent conditions, anchoring it in the cytoplasm and eventually targeting for degradation in proteosomes [95]. Oxidation or alkylation by electrophiles of the cysteine residues in Keap1 terminates its sequestration from the Nrf2, allowing the latter to accumulate and translocate to the nucleus wherein it activates transcription of cytoprotective genes [94]. Thus, Nrf2-Keap1 is considered the main signaling pathway activated to restore redox balance [96].

### 3.6. Multidimensional Role of FOXOs in Cell Fate

There is evidence that PERK may significantly increase activity of FOXOs via its phosphorylation or, conversely, indirectly decrease it via Akt. PERK acts on Akt via phosphorylation of diacylglycerol (DAG) to increase its activity, which in turn downregulates PERK [97,98,99]. Furthermore, phosphorylation of FOXOs might also be triggered by JNK, AKT, AMP-activated protein kinase (AMPK) or mammalian Ste20-like kinase (Mst1), all of which become activated under conditions of oxidative stress and nutrient deprivation [100,101]. Translocation of FOXOs into the nucleus orchestrates the expression of the target genes such as *Kinase Inhibitor Protein 1* (*KIP1*), *Growth Arrest And DNA Damage Inducible protein* (*GADD45*), *DNA Damage Binding protein 1* (*DDB1*), *Bcl-2-like protein 11* (*BIM*), *Fas ligand* (*FasL*), *Manganese-dependent superoxide dismutase* (*MnSOD*), and *catalase*. It has been reported that above-mentioned genes play a critical role in cell cycle regulation, resistance to oxidative stress, DNA damage repair, and apoptotic cell death [102].

Another indirect role of FOXO includes integrating upstream UPR signals with the transcriptional machinery in order to decrease global translation and increase the levels of chaperones and ERAD components [103]. Despite the general pro-survival role of FOXOs, the pro-apoptotic signals in response to ER stress may also be triggered in certain circumstances. The decision whether stress-resistance genes or pro-apoptotic factors (below or over the threshold, respectively) are activated to execute survival or apoptotic responses depends on two key factors: The intensity of the stimulus as well as the type of the affected cell [104]. Various proliferation suppressors such as similar to mothers against decapentaplegic (Smad) or p53, representing FOXOs partners influenced by their activity, also appear to be critical in cell fate determination [105,106].

### 3.7. CHOP Protein and JNK as the Key Factors in Apoptosis Induction

There is abundant evidence that CHOP is responsible for regulation of numerous genes implicated in cell migration, proliferation, and survival [107]. Under severe, irremediable ER stress conditions, PERK/elF2α/ATF4/CHOP and IRE1α/TRAF2/c-Jun N-terminal inhibitory kinase/apoptosis signal-regulating kinase/JNK (IRE1α/TRAF2/JIK/ASK1/JNK) signaling pathways may initiate apoptosis [108,109,110]. CHOP, which in turn downregulates anti-apoptotic, mitochondrial Bcl-2 protein [111] and hyperoxidizes the ER environment via ERO1α induction, is considered one of the main pro-apoptotic ER stress mediators, together with caspase-12. Interestingly, it is not the only death pathway uncovered thus far. ERO1α, besides supporting disulfide bond formation primarily, causes hydrogen peroxide leakage into the cytoplasm [112]. This generates calcium ions flow through the inositol-1,4,5-trisphosphate receptor 1 (IP3R1) channel, directly leading to the activation of calcium/calmodulin-dependent kinase II (CaMKII) and hence the initiation of the four major pro-apoptotic cellular events, including: increased mitochondrial calcium uptake, induction of JNK-mediated Fas (Fas) antigen, pro-apoptotic signal transducer, and activator of transcription 1 (STAT1) as well as NADPH oxidase subunit 2 (NOX2) with consequent ROS generation. Excessive ROS accumulation in turn amplifies CaMKII activation and CHOP expression in a positive feedback loop [7,113,114,115]. Furthermore, during prolonged ER stress conditions, both PERK- and IRE1α-mediated signaling pathways may converge on CHOP since its activity is increased via phosphorylation by p38, a commonly known substrate of IRE1α-recruited apoptosis signal-regulating kinase ASK1 [116,117,118,119,120,121]. Remarkably, more recent data have revealed that the factor which determines the switch from cell survival to apoptosis is the timing of IRE1α and PERK signaling pathways rather than exclusive activation of a single branch of UPR or switch from one branch to another [122].

The newest data has reported that the role of JNK is pivotal in the mediation of ER stress-induced apoptosis upon prolonged expression of IRE1α and CHOP. It constitutes a downstream effector of the CHOP-CaMKII and IRE1α-TRAF2 signaling pathways, respectively (the latter exerts paradoxically opposite effect on cell survival compared to cytoprotective IRE1α-XBP1) [110,115,123]. JNK may downregulate anti-apoptotic proteins such as BCL-2, BAD or BAX [124,125], with parallel activation of pro-apoptotic BID, BIM and Bcl-2-modifying factor (BMF), all of which belong to so-called BH3-only family [126,127]. JNK-mediated phosphorylation causes BIM to detach from its inhibitory dynein motor complexes and insert into the outer mitochondrial membrane (OMM) to promote cytochrome C (cyt c) release with subsequent caspase activation [127,128]. However, it should be mentioned that UPR-mediated JNK signaling can be described as biphasic—when activated immediately, in its early phase, it is antiapoptotic, whilst in late phase it promotes cell death. Such opposing effects of JNK on cell viability exist either in ER stress or other types of stress response [129].

Sensitivity of apoptosis is controlled by the ratio of anti-apoptotic (BCL-2, B-cell lymphoma-extra-large BCL-XL, and MCL-1) to pro-apoptotic (BAX, BAK, and BH3-only proteins) BCL-2 family members [130,131,132]. The proteins are physically interacted with certain UPR components, being tightly modulated by action of each branch on different levels. For example, upon severe, long-termed ER stress conditions, PERK/ATF4 branch of the UPR signaling pathway plays a key role during induction of the BH3-only proteins such as activator of apoptosis HARAKIRI (PUMA) and p53 up-regulated modulator of apoptosis (NOXA). Moreover, above-mentioned event also evokes upregulation of CHOP, which directly enhances BIM, PUMA and BAX expression and, on the other hand, downregulates BCL-2 and MCL-1 expression. Interestingly, it has been reported that ATF6 indirectly inhibited MCL-1 expression in a myoblast cell line. According to the output, the effects of IRE1α on the BCL-2 family may vary from anti-apoptotic (XBP1s-mediated, with exclusive expression of BAK, BIM and PUMA) to pro-apoptotic (TRAF2-JNK-mediated) [71,130].

**Figure 2:**
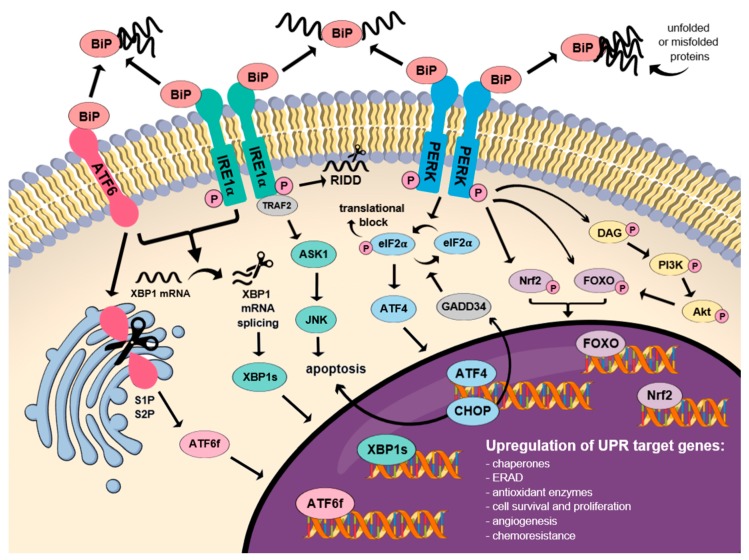
Three major branches of the Unfolded Protein Response (UPR) signaling pathway and their interconnections. Under endoplasmic reticulum (ER) stress conditions, damaged proteins accumulate within the ER lumen and bind to binding immunoglobulin proteins (BiPs), which in turn dissociate from transmembrane complexes with activating transcription factor 6 (ATF6), inositol-requiring enzyme-1α (IRE1α) and the protein kinase R (PKR)-like endoplasmic reticulum kinase (PERK) in attempt to refold the proteins. As a result, three branches of the UPR become activated and trigger adequate stress responses. ATF6, after cleavage in Golgi apparatus, enters the nucleus to activate cytoprotective genes. It also upregulates X-box binding protein 1 (XBP1) mRNA, which is subsequently spliced by IRE1α to enhance the pro-survival effect. The main downstream target of PERK, eukaryotic translation initiation factor 2α (eIF2α), inhibits the global protein synthesis, with the exclusive translation of specific mRNAs such as activating transcription factor 4 (ATF4). Both nuclear factor-like 2 (Nrf2) and forkhead box protein O1 (FOXO) proteins, directly phosphorylated by PERK, are responsible for antioxidant and cell survival mechanisms. Additionally, PERK may act on FOXO indirectly via phosphoinositide-3 kinase/protein kinase B (PI3K/Akt) signaling pathway. Apoptosis may be induced either by IRE1α, via c-Jun N-terminal kinases (JNKs) signaling pathway, or by PERK-mediated C/EBP homologous protein transcription factor (CHOP).

Once the intensity of ER stress conditions reaches its threshold, ER-associated BAX and BAK proteins change their conformation and oligomerize, resulting in an intracellular calcium leak followed by, respectively, m-calpain (or calpain-1 and -2) and procaspase-12 activation. Those proteins associated with mitochondria induce cleavage of caspase-7, representing a parallel pathway of caspase activation [133,134]. The cleavage of ER-localised procaspase-12 causes its subsequent transformation into caspase-12, pivotal in stress-mediated apoptosis due to its capability to inhibit NF-κB, a transcription factor involved both in the immune response and apoptosis [23,135,136] (Figure 2).

## 4. Molecular Basis of UPR in Cancer Development

### 4.1. ER Stress-mediated Activation of the UPR Signaling Pathway in Cancer Cells

ER stress and UPR signaling pathway activation have an essential impact on all stages of cancer progression. It has been reported that ER stress activation constitutes a characteristic feature of highly aggressive cancers [137,138]. For instance, activation of the UPR has been confirmed in prostate cancer, since the UPR-related factors were markedly increased. UPR induction was correlated with malignant progression and worse prognosis of prostate cancer. Patients with positive UPR signaling factors have been characterized by shorter survival duration [139]. During the metastatic stage, the epithelial to mesenchymal transition (EMT) promotes cancer progression, enhances its drug resistance and creation of significantly more invasive phenotype. It has been reported that ER stress activation intensifies the EMT both by the IRE1α- and PERK-mediated UPR signaling pathways [140,141]. Persistent ER stress has also been reported in B cell malignancy multiple myeloma (MM), that constitutes highly secretory cancer due to the enhanced synthesis of immunoglobulins [142]. Additionally, ER stress and UPR activation have been demonstrated in breast cancer, wherein they promote malignancy and resistance to commonly used treatment modalities [68]. It has also been reported that activation of the PERK-dependent UPR signaling pathway promotes CRC progression. PERK plays a pivotal role in tumor cell adaptation to hypoxic stress by regulating the translation of angiogenic factors necessary for the development of functional microvessels [143]. Thus, the above-mentioned research underlines a prominent role of ER stress and UPR signaling pathway activation in cancer initiation and progression.

### 4.2. BiPs as the Markers of the UPR Signaling Pathway Activation in Cancer

An elevated level of BiP constitutes one of the major markers of UPR signaling pathway activation, since BiP level is regulated by all of the UPR signaling branches [144,145,146]. During the past decade, common and distinctive functions of GRP78 in cancer have been discovered and currently there are multiple lines of evidence confirming correlation between enhanced GRP78 expression and tumor aggressive growth as well as its invasive and metastatic properties [147]. It has been demonstrated that the level of BiP chaperones was significantly increased in various tumor types, especially during cancer metastasis, in comparison with normal cells in which BiP chaperones were weakly expressed, or absent [148].

It has been reported that an increased level of GRP78 promotes almost every step of carcinogenesis and alters responsiveness to anticancer drugs. There has been found a correlation between GRP78 and the adhesion molecule N-cadherin (N-cad), a key mediator in the adhesion of MM or metastatic prostate cancer cells with the bone microenvironment [149]. Besides, BiP overexpression has also been correlated with poor clinical prognosis and weak response to treatment in patients suffering from rectal cancer in a clinical trial [150]. In tumorigenesis, BiP revealed their pro-survival and pro-metastatic functions with PI3K/Akt signaling pathways being key factors in these hallmark processes [27]. Another study implicated that there is an interaction between the cell migration-inducing protein (CEMIP) and GRP78, which provides protective adaptations of cancer cells under hypoxic conditions. Mechanistically, CEMIP resides in the ER where it mediates the activation of the GRP78 promoter, upregulates its transcript and protein levels. As a result, both proteins are upregulated in tumor cells, where they facilitate tumor growth, drive progression and metastasis [151]. Furthermore, a study by Mhaidat et al. (2016) has revealed high levels of GRP78 both in CRC cell lines and CRC tissues isolated from patients. Inhibition of GRP78 evokes enhanced sensitivity of CRC cells to chemotherapeutic agents [152]. Numerous studies have suggested that GRP78 is implicated in chemoresistance to 5-fluorouracil (5-FU) and oxiplatin in CRC cells, given that the silencing of GRP78 improved response to treatment and effectively suppressed tumor growth via the induction of apoptosis [153,154,155]. Also, a significantly elevated level of GRP78 has been confirmed in breast cancer tissue in comparison with normal tissue, which underlines a pivotal role of GRP78 in breast cancer development and progression [156]. Moreover, it has been confirmed that GRP78 may induce stemness in pancreatic and head and neck cancer, thereby contributing to their aggressive properties such as increased clonogenicity or self-renewal [157,158]. The transcripts and protein levels of GRP78 are significantly elevated in head and neck squamous cell carcinoma. It has been demonstrated that overexpression of GRP78 enhances head and neck cancer malignancy, whereas GRP78 knockdown promotes cancer cells apoptosis [159].

Moreover, it should be mentioned that the targeting of GRP78 with novel therapeutic agents appeared to be effective both in in vitro and in vivo studies, wherein it suppressed tumor growth. These findings may contribute to the development of effective, antineoplastic treatment strategy in the future [160,161,162]. On the contrary, macrolide antibiotic brefeldin A (BFA) exhibited antitumor activity in CRC cells both in in vitro and in vivo models via inducing GRP78 expression and increasing the GRP78/Akt signaling pathway, which ultimately activated a complete BiP-regulated autophagic flux [163].

### 4.3. IRE1α/XBP1-dependent Regulation of Tumor Growth

A large body of evidence suggests that either the upregulation [164,165] or downregulation [166] of IRE1α promotes tumor growth in certain circumstances, which may imply its dual role in carcinogenesis. Moreover, a study by Lhomond et al. [167] demonstrated two opposing effects of IRE1α on progression of primary brain tumor, glioblastoma multiform (GBM), depending on activation of XBP1s or RIDD pathways. On the one hand, XBP1s sustains pro-tumorigenic signals, promotes angiogenesis and macrophage recruitment, while, on the other hand, RIDD suppresses angiogenesis and cell migration. Accordingly, tumors demonstrating high XBP1s but low RIDD expression were correlated with reduced survival in patients than the ones with opposite features [167]. Nevertheless, the inhibition of IRE1α has already demonstrated positive therapeutic outcomes in Ewing′s sarcoma [168], MM [169], acute myeloid leukemia (AML) [170] as well as triple-negative breast cancer (TNBC) [165]. Activation of the IRE1-mediated arm of the UPR in breast adenocarcinoma has been confirmed by immunohistochemical analysis which demonstrated significantly elevated levels of XBP1 in 90% of analysed samples [156]. Study by Logue et al. [165] has also shown intensive splicing of XBP1 in breast cancer, indicating the IRE1α activity in these cells. Besides, the ratio of spliced to total XBP1 was highest in samples collected directly from breast cancerous tissue as compared to luminal samples or normal tissue surrounding a tumor. Interestingly, the inhibition of IRE1α activity by MKC8866 in breast cancer cells triggers decreased production of pro-tumorigenic factors such as Interleukin 6 (IL-6), Interleukin 8 (IL-8), chemokine (C-X-C motif) ligand 1 (CXCL1), transforming growth factor-β2 (TGFβ2), and granulocyte-macrophage-colony-stimulating factor (GM-CSF) as well as enhancing breast cancer cell response to chemotherapy [165].

XBP1 splicing itself may directly lead to carcinogenesis, since its increased expression has been found in numerous haematological malignancies and solid tumor types; likewise it is associated with more malignant phenotypes and poor survival [27,171,172,173,174,175]. Indeed, it has been confirmed that hyperactivation of XBP1 together with HIF1α is essential for the tumorigenicity and progression of TNBC as well as correlated with poor prognosis in TNBC patients [60,61]. Moreover, the activation of the IRE1α/XBP1/cMyc signaling pathway has been shown to be a key regulator in Natural Killer (NK) cell immunity against viral infection and tumors in vivo [176]. Moreover, the newest data has indicated that adaptive signaling via IRE1α-XBP1 preserves self-renewal of haematopoietic stem cells (HSC), protects them from ER stress-induced apoptosis, and promotes pre-leukaemic clonal dominance [177]. Curiously, ovarian cancer cells have been shown to be capable of inducing the IRE1α-XBP1 arm in T cells to decrease their anti-tumor activity via suppressing mitochondrial respiration and interferon gamma (IFNγ) production [178]. Additionally, in a prostate cancer in vitro model, it has been confirmed that cell proliferation resulted from IRE1α-driven cyclin A1 expression, regulated by XBP1 splicing [179]. Furthermore, IRE1α-XBP1 axis has been proven to promote doxorubicin (DOX) and paclitaxel (PTX) resistance in TNBC, while in parallel it led to resistance to tamoxifen (TAM) in ESR1+ cell line, presumably induced by NF-κB [60,63,180].

Recently, the correlation between XBP1 signaling and IL-6 has also been investigated. New findings suggest that IRE1α-XBP1 signaling promotes development of tumors such as hepatocellular carcinoma (HCC) or melanoma via upregulation of IL-6-driven Janus kinase-signal transducer and activator of transcription 3 (JAK-STAT3) signaling pathway. It has been confirmed that XBP1s binds to the IL-6 promoter to activate its expression, and IL-6 in turn activates JAK-STAT3 signaling in an autocrine/paracrine manner. Additionally, protein expression levels of XBP1s and IL-6 were significantly elevated and positively correlated with each other in tumor tissues [181,182]. More recent data has also revealed that IRE1α-XBP1 axis regulates radioresistance via activation of IL-6, since silencing of the pathway enhanced radiation-induced apoptosis in papillomavirus-negative oropharyngeal carcinoma [183].

### 4.4. Cytoprotective and Pro-tumorigenic Role of ATF6

Due to the general pro-survival role of ATF6, its significantly increased level has been widely shown to occur in various cancer types [184,185,186]. Interestingly, a high level of ATF6 has been strictly correlated with cancer metastasis and recurrence [187]. In a CRC model, the activation of XBP1 and ATF6 resulted in reduction of stemness and proliferation of cancer cells via the crossactivation of the PERK-eIF2α signaling pathway [188]. On the contrary, higher expression of ATF6 was found in lesions undergoing pre-cancerous atypical change in CRC [189], as well as it was correlated with poor prognosis in patients suffering from CRC [190,191]. Thus, the actual role of ATF6 in the molecular mechanism of CRC development needs to be further elucidated. It has also been confirmed that ATF6 possesses pro-oncogenic functions and may contribute to hepatocellular carcinogenesis [192,193]. Furthermore, treatment of human hepatoma HepG2 cells with a novel selective ATF6 inhibitor, melatonin, sensitized cells to ER stress-induced apoptosis, and thus provided promising results [184]. In another study by Sicari et al. (2019), the activity of ATF6 appeared to be essential for viability and invasion phenotypes in TNBC cells [194]. ATF6 has also been shown to induce resistance, and thereby decrease the efficacy of conventional therapy in glioblastoma [195] and osteosarcoma (OS) [196]. Besides all the above mentioned reports, however, ATF6 has no obvious paradoxical outcomes confirmed in contrast to PERK or IRE1α [27].

### 4.5. Paradoxical Outcomes of PERK Activation within Cancer Cells

PERK, which is a serine/threonine ER kinase, is the most rapidly activated among all of the three branches of the UPR signaling pathway, followed by ATF6 and IRE1α sequentially [197,198,199,200]. PERK basically serves as a sensor of excessive oxidative processes within the cells and a barrier to malignant growth in certain circumstances. In comparison with normal tissues, the level of p-PERK appears to be notably higher in tumor cells, presumably due to the exploitation of the PERK-dependent signaling pathway to progress and survive in harsh microenvironments [60,201,202]. The PERK-dependent UPR signaling pathway is strictly correlated with cancer invasion and metastasis on the molecular level and therefore it is being thoroughly investigated worldwide for development of a novel anti-cancer treatment strategy [137,203].

There is numerous data on significant impact of p-eIF2α on patient prognosis in multiple types of cancer. Interestingly, markedly elevated level of the p-eIF2α has been previously demonstrated, as compared to noncancerous cells, in cancer cells including bronchioloalveolar carcinoma, Hodgkin’s lymphoma, gastrointestinal carcinoma as well as benign and malignant melanocytic and colonic epithelial neoplasms [204,205,206,207]. Moreover, data reported by Guo et al. [208] revealed that evaluation of the cellular p-eIF2α level may have a clinical application, since the level of p-eIF2α is significantly enhanced in breast cancer cells in comparison with peritumor tissue. Above-mentioned study has suggested that p-eIF2α may inhibit a tumor invasive growth in TNBC, that represents an extremally aggressive breast cancer subtype with a poor prognosis for patients, as well as enhanced expression of p-eIF2α is strictly correlated with weaken tumor invasion of lymph nodes. Thereby, in this case, p-eIF2α may constitute an important TNBC biomarker as well as attractive target for the TNBC treatment [208].

However, the role of the eIF2α in cancer initiation and progression is not fully understood and needs further investigation, since several studies demonstrated that p-eIF2α may also prevent tumorigenesis. Decreased level of p-eIF2α has been reported in human OS, which constitutes the most common malignant tumor in children and young adults, as compared to noncancerous tissue [209]. Moreover, the newest data reported by Wang et al. (2019) demonstrated, both in in vitro and in vivo experimental models, that CYT997-mediated intensity of the PERK/p-eIF2α/CHOP signaling pathway may induce apoptotic OS cell death and thereby decrease tumor growth [210]. Besides, the most recent data has indicated that PERK/eIF2α/ATF4/CHOP signaling pathway may be involved in the HCC, the most common primary liver cancer, development and progression. Pterostilbene (PT) treatment of HCC cells resulted in enhanced induction of the ER stress conditions and subsequently increased expression of the UPR markers including BiP, PERK, eIF2α, ATF4 and CHOP, leading to cell cycle arrest and HCC cells death [211]. Furthermore, Wang et al. [212] have investigated the involvement of the PERK-dependent UPR signaling pathway in human lung adenocarcinoma initiation and progression as well as its potential role in the development of a novel treatment strategy against lung cancer. Treatment of A549 cells with Paraquat evoked A549 apoptotic cell death which was strictly associated with the regulation of the UPR signaling pathways, especially PERK/eIF2α-dependent one [212]. A study by Del Vecchio et al. (2014) has demonstrated that cells treated with the GSK2606414 inhibitor revealed PERK to be in fact linked to higher tumor grades and worse patient survival [213]. Conversely, in a separate study carried out by Gupta et al. (2009), PERK-deficiency resulted in smaller tumor size, impaired angiogenesis, reduced viability and metastatic spread during hypoxia, probably caused by the decline of p-eIF2α and ATF4 [214]. PERK-knockdown cells also exhibited reduced Nrf2 activity and, in consequence, cell cycle arrest, notably observed in esophageal and breast carcinomas. This could be explained by the fact that the Nrf2 ablation sequentially drives impaired regeneration of intracellular antioxidants, accumulation of ROS, oxidative DNA damage, cell cycle checkpoint activation, and eventually significant attenuation of tumor growth [215]. Thus, PERK loss basically sensitizes tumor cells to oxidative DNA damage, but, on the other hand, it appears to be pro-tumorigenic due to induction of extended genotoxic stress, accompanied by mutational DNA damage response (DDR) inactivation. This happens because long-term DNA damage can override the DNA damage checkpoint, causing genomic instability and thereby increased frequency of spontaneous mutations, capable of promoting neoplastic growth.

Indeed, in several studies it was observed that PERK deletion deregulates cellular morphogenesis, which leads to hyperplastic growth both in vitro and in vivo, presumably due to increased genomic instability and tumor susceptibility. Such changes, however, can in most cases be observed over the lifetime of the organism. For instance, spontaneously formed mammary tumors have been demonstrated in aged PERK-null mice, compared to used controls [216], as well as in the separated experiment MCF10A cells expressing PERKΔC favored formation of the same tumor type [217]. Mentioned reports imply the suppressive role of the PERK-eIF2α signaling pathway, connected with dysregulation of cyclin D1 expression [218,219]. Interestingly, in another study by Pytel et al. [220], it was proven that PERK^+/−^ deficient mice were permissive to develop BRAF-dependent melanoma, whereas complete PERK inhibition (PERK^−/−^ genotype) appeared to be tumor suppressive. This clearly indicates the complex role of the PERK activation as the kinase may act both as pro- and anti-tumorigenic, depending on the gene dosage. Retention of one allele of PERK appears to be essential for tumor progression, in contrast to the deletion of two alleles, which demonstrates diametrically opposing results [220]. This phenomenon can be explained by overexpression of cyclin D1 and repression of CHOP translation in PERK^+/−^ mutant cells [220,221]. Oddly enough, a study by Bobrovnikova-Marjon et al. (2010) revealed that lack of PERK does not acutely affect normal tissue, as regards mammary epithelial cells, supposedly due to normal homeostatic proliferation, not inducing significant mitochondrial ROS generation [216]. Conversely, PERK-null mice exhibited symptoms similar to those seen in human Wolcott–Rallison syndrome, associated with ER dysfunction in major secretory cells of the specific tissues, resulting in skeletal, pancreatic, and growth defects [222]. It is for this reason that, when it comes to insulinomas, ablation of PERK was described to decrease tumor size, inhibit cell proliferation and angiogenesis [214]. In contrast to the previous results, PERK-driven p38 has been found to induce dormancy in squamous cell carcinoma, similarly to pharmacologically-activated PERK, which suppressed tumor growth [223]. In addition, knockdown of the PERK/ATF4/LAMP3 signaling pathway, which is mobilized via radiotherapy, downregulates the DDR and sensitizes tumor cells to radiation [224].

Interestingly, the intensity of PERK-mediated UPR signaling pathway may also be measured by the level of its main substrates including, except for eIF2α, Nrf2. According to the newest reports, the role of Nrf2, as one of the major PERK substrates, in carcinogenesis can be described as dual-protective at early stages or, conversely, detrimental and pro-oncogenic at later stages of the disease. At lower levels, Nrf2 suppresses tumorigenesis via eliminating carcinogens, ROS and other DNA-damaging agents. On the other hand, aberrant activation of Nrf2 found in cancer cells helps them to withstand high levels of ROS and avoid apoptosis [225]. Many studies indeed have suggested that upregulation of Nrf2 in a variety of tumor types is associated with cancer progression, chemoresistance (including multidrug resistance) and radioresistance [29,226,227,228,229,230]. It has been reported that PERK is responsible for Nrf2-promoted resistance to PTX and DOX in differentiating cancer cells, but in some circumstances it may trigger drug-induced cell death [213].

Thereby, both activation and inactivation of the PERK-dependent signaling axis seem to have paradoxical effects on various stages of carcinogenesis. Thus, the exact role of PERK remains still contradictory and unclear, whether it upregulates tumorigenesis or, conversely, suppresses it. Nevertheless, inhibition of the PERK-dependent UPR signaling pathway seems to be an effective strategy not only as regards cancer cells elimination, but also regarding many other ER stress-dependent disorders like diabetes, neurodegenerative, or heart diseases [75].

### 4.6. The Role of ATF4 in Cancer Initiation and Progression

It has been proven that transcription of many essential pro-tumorigenic genes, involved in cancer metastasis, angiogenesis, and drug resistance, is ATF4-driven. Interestingly, ATF4 downstream effectors ULK1 and LAMP3 were found to be overexpressed in breast cancer and correlated with more advanced cancer stage [83,84,231]. It is not surprising then that increased expression of ATF4 was found in several solid tumor types [80,232,233,234]. Accordingly, it was identified that ATF4 overexpression facilitates progression and fosters the malignancy of tumors via increasing their proliferation, vessel growth, cell migration, and metastasis [79,234,235,236,237].

In the case of OS, it was suggested that such upregulation of *ATF4* found both in cell lines and patient clinical samples was due to a positive feedback loop between ATF4 and metastasis associated protein/histone deacetylase 1 (MTA1/HDAC1) axis. Moreover, in a mouse xenograft model of ATF4-overexpressing OS cells, tumors grew faster and had 90% greater weight than in controls [234]. A recent study by Shi et al. [233] also revealed that not only PERK, but also ATF4 activity was significantly elevated in CRC cells demonstrating heightened resistance to 5-FU, a chemotherapeutic for the first-line treatment of patients with advanced CRC. This finding indicates the involvement of PERK/ATF4 axis in chemoresistance promotion. The inhibition of PERK with a specific GSK inhibitor synergized with 5-FU resulted in suppressed tumor growth in mice, thereby we can conclude that the silencing of the PERK-ATF4 branch of the UPR signaling pathway greatly sensitizes CRC cells to 5-FU.

The fact that overexpression of ATF4 aggravates malignant phenotypes has also been confirmed in the case of high-grade gliomas (HGG), wherein it evokes increased proliferation, angiogenesis, and tumor cell migration [79]. According to the newest data, ATF4-driven expression of family with sequence similarity 129 member A (FAM129A) is required for prostate tumorigenesis. *FAM129A*, as one of the ATF4 target genes, was also confirmed to regulate both PERK and eIF2α in a feedback loop, thereby differentially channeling the outcome of the UPR activation [235]. On the contrary, it was also found that the knockdown of ATF4 in two endometrial cancer (EC) cell lines suppressed tumor growth in vivo but had no impact on cell proliferation in vitro. The above-mentioned event may be explained by the fact that ATF4 mediates the expression of chemokine (C-C motif) ligand 2 (CCL2), which promotes subsequent recruitment of macrophage and thereby contributes to tumor growth. As it has been found in experimental model, in xenograft tumors derived from ATF4-knockdown cells, the macrophage infiltration was significantly reduced [237].

### 4.7. A critical Role of FOXO Proteins in Carcinogenesis

During carcinogenesis, chromosomal translocations or deregulated signaling pathways may lead to loss-of-function mutations of FOXOs, which are highly desirable to enable tumor progression [238]. In fact, numerous studies have revealed that FOXOs may be implicated not only in cancer progression, but also in resistance and sensitivity to treatment by promoting DNA repair, growth arrest, and, on the other hand, apoptotic genes expression [239,240]. As a couple of examples, there has been found a link between the reduced expression of FOXO1 and resistance to conventional chemotherapeutic agents, which was observed in ovarian [241], nasopharyngeal [242] or esophageal squamous cell carcinoma [243]. By contrast, FOXO3 was shown to sensitize cancer cells to chemotherapy by mediating cytotoxic and cytostatic effects of cancer therapeutics such as cisplatin (CDDP) [244,245,246,247]. Moreover, a recent study by Alasiri et al. [248] also revealed that FOXO3 was significantly downregulated in drug-resistant breast cancer cells as one of the adaptive mechanisms acquired by cancer. Oddly enough, cells used in the experiment demonstrated low expression, but high activity of PERK simultaneously and were also specifically sensitive to PERK inhibition [248]. There is also evidence that FOXOs represent a downstream target in the PI3K/Akt signaling pathway which may be engaged, together with increased expression of FOXOs, in drug resistance [102]. Nevertheless, the significance of FOXOs in deciding cancer cell fate cannot be simplified and ought to be further elucidated, given that they undergo multiple post-translational modifications [249].

### 4.8. Multiple Functions of Apoptotic Proteins in Deciding Cancer Cell Fate

In several tumor types such as breast, gastric, and cervical cancer, CHOP has been reported to act as a key mediator in the ER stress-related cell death induced by Ilamycin E [111], kaempferol [118] and fucoidan [250], respectively. When it comes to HCC, however, the actual role of CHOP seems to be much more complex. Recent data has indicated that CHOP favors ER stress-induced apoptosis via autophagy inhibition in a HCC model in vitro [251], whereas in another study it contributed to development of HCC via promoting cell death, inflammation, fibrosis and compensatory proliferation (attributes characteristic for chronic liver diseases) [252]. Besides, CHOP is also considered a major negative regulator of the effector tumor-reactive CD8+ (cytotoxic) T cells, and hence overexpression of CHOP contributes to their dysfunction [253].

In a breast cancer model, loss of JNK was proven to cause genomic instability and rapid tumor formation, which may suggest that defects in JNK signaling act as driver mutations in promoting tumor initiation [254]. In separated studies, the high activity of JNK favored tumor initiation, metastasis and was linked to poor clinical outcome in mammary tumors in vivo [255], as well as it drove TNBC tumorigenesis by promoting cancer stem-like cell (CSC) phenotype through the Notch1 signaling pathway [256]. Although being primarily a pro-apoptotic factor, JNK has also been suggested to cause resistance to CDDP-induced apoptosis in human tumors [257].

For normal functioning of the cell, the equilibrium between pro- and anti-apoptotic Bcl-2 family members is pivotal. In cancer cells, the expression of mentioned proteins is often deregulated, which prevents them from apoptosis, facilitates their proliferation and resistance to therapy [258,259]. Therefore, drugs mimicking the action of BH3-only proteins via activation of BAX/BAK have emerged as a novel therapeutic anti-cancer strategy. Recent data has demonstrated promising results after treatment with BH3-mimetics cells representing different malignancies such as chronic lymphocytic leukemia (with Venetoclax being officially approved for the treatment) [259,260], prostate cancer [261,262] or small-lung cancer [263]. However, some therapeutic problems undermining the clinical utility of BH3-mimetics have also been reported [264].

A recent study demonstrated that overexpression of caspase-12 mediated NF-κB activation via degradation of the nuclear factor of kappa light polypeptide gene enhancer in B-cells inhibitor, alpha (IκBα), which resulted in nasopharyngeal carcinoma (NPC) cells invasion [265]. Moreover, caspase-12 might be activated via IRE1α-recruited dissociation of procaspase-12 from TRAF2. The processing of other caspases (-2 to -9 in particular) has also been shown to both initiate and execute apoptosis under ER stress conditions [266] 

## 5. The Role of Reactive Oxygen Species in Carcinogenesis

Electrophilic and highly reactive oxygen free radicals commonly termed ROS are considered one of the most pervasive and potent threats to cell homeostasis. These include, among others, singlet oxygen, superoxide anion, hydrogen peroxide, hydroxyl and peroxy radicals, as well as nitric oxide, a relevant second messenger [132]. All of them are mainly originated from endoplasmic NADPH oxidase (NOX) and mitochondria, produced therein via normal metabolic processes although they may be induced by numerous external factors as well [267]. Above-mentioned signaling molecules are poised to play a key role in cell fate determination, regulation of multiple physiological functions, defense mechanisms and intracellular pathways concurrently. However, excessive ROS generation (both endogenous and exogenous) may directly evoke redox imbalance and consequential cellular and tissue damage [94]. This occurs mainly under stress conditions and is present in several types of diseases, when the equilibrium between pro-oxidant species and antioxidant defense mechanisms (GSH and ROS-metabolizing enzymes—catalase, peroxidase, GPx, SODs etc.) becomes distorted [268].

Overwhelming ROS levels can cause damage to almost every class of biological macromolecules, resulting in ER dysfunction, unfolded and misfolded protein accumulation and consequently induction of oxidative stress. The aforementioned event may lead directly to significant cell function impairment, or even evoke both apoptotic and necrotic cell death. There is ample evidence that once ROS overproduction takes place, a series of pro-apoptotic signaling pathways, including UPR, become immediately activated [267]. Finally, ROS overload stimulates PERK-dependent phosphorylation of the Nrf2 transcription factor, whose target genes are involved in antioxidant defense through enzymes of GSH synthesis and HO-1 [269]. Besides, under such circumstances, cytosolic calcium overload, followed by the upregulation of the cytoplasmic cysteine proteases calpain-1 and -2, both associated with calcium metabolism, has been reported. Accordingly, the downregulation of BCL-2 with concomitant upregulation of Bax, caspase-12, and other apoptosis-related proteins, follows.

In addition, oxidants and antioxidants, besides deciding cell fate, are also capable of determining the mode of cell death [219,268]. For instance, hydrogen peroxide (H_2_O_2_) supports cyt c release from the mitochondria, that activates pro-apoptotic nuclear transcription factors (e.g., p53, NF-κB, and activator protein 1 AP-1), and finally upregulates the Fas-FasL ligands, which leads to the activation of downstream caspases, such as caspase-8 [270,271].

Several lines of evidence have confirmed that there are two possible mechanisms responsible for the molecular switch of the apoptotic cell death to necrosis during oxidative stress: The inactivation of cellular caspases (via oxidation, thiol alkylation or S-nitrosylation of an active-site cysteine) and an increase in Ca^2+^ with subsequent decrease in ATP cellular levels (hallmarks of mitochondrial dysfunction, correlated with opening of the mitochondrial permeability transition pore (MPTP)) [132,272,273,274,275] Thus, imbalanced ROS and electrophile overproduction are considered to be critical promoters in onset and progression not only of cancer, but also of several neurodegenerative disorders, such as Alzheimer′s disease (AD) or Parkinson′s disease (PD).

**Figure 3:**
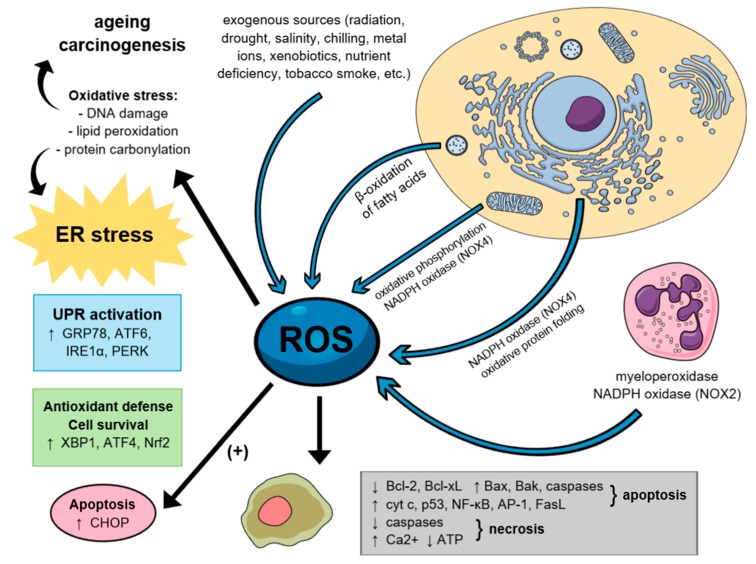
Biological origin of free radicals and their effect on cell viability. Reactive Oxygen Species (ROS) are generally derived both from intrinsic factors (enzymatic reactions physiologically conducted in subcellular organelles) and from the numerous extrinsic ones. Although ROS evidently remain essential for normal functioning of the cell, including immune response or redox signaling, their overload may prove harmful and cause significant damage. Ultimately, it may lead to execution of apoptotic or necrotic cell death or even drive tumor growth. Unfolded Protein Response (UPR) signaling pathway is closely linked to ROS-induced oxidative stress and it becomes activated to address cellular redox imbalance.

As mentioned before, cancer cells exhibit persistently elevated level of ROS, which is mainly due to high metabolic rates, mitochondrial dysfunction and activation of NOX in cellular membranes. They possess increased antioxidant ability simultaneously in order to compensate for ROS overload. ROS play a key role at almost every stage of carcinogenesis—primarily, they can cause oxidative damage to DNA including activation of oncogenes such as Ras, Bcr-Abl and c-Myc [276]. For instance, H2O2 emerges a key mediator in inducing both cancer initiation, via activation of PI3K-Akt-mammalian target of rapamycin (PI3K-Akt-mTOR) axis, and metastasis, via activation and stabilization of HIF1α during hypoxia [277,278]. Both mentioned mechanisms have also been shown to enhance cancer cell migration and invasion [279]. Nowadays, ROS attract particular interest as a potential target in cancer cell treatment since they possess an ability to trigger apoptosis via DNA damage. Furthermore, agents with ROS-generating potential have already been reported to be effective against CRC cells [267] (Figure 3).

## 6. Innovative Drugs Depending on Oxidative Mechanisms

Three major ATP-competitive PERK inhibitors have been discovered so far: GSK2606414, GSK2656157, and AMG’44 [280]. Although AMG’44, in comparison with over 300 kinases tested, is said to be the most selective for PERK, it has not been tested on various cancer models yet. The most familiar small-molecule PERK inhibitor, GSK2606414, which is said to be the first one with high specificity for this kinase, was successfully tested on HT-29 colon cancer cell culture and proven to reduce breast cancer cells metastasis in vivo [281,282]. It was also found to sensitize human mammary epithelial cells (HMLE) and TNBC cell cultures to chemotherapeutic agents such as PTX and DOX [213]. However, GSK2606414 also evoked significant side effects, since its high cytotoxicity resulted in weight loss and hyperglycemia in in vivo animal model, which may have been due to the undesirable inhibition of PERK within the pancreatic cells [283,284,285]. Study by Atkins et al. [284] demonstrated that administration of GSK2656157 exhibited high anti-tumor activity, since it evoked dose-dependent inhibition of multiple human tumor xenografts in mice. However, despite the GSK2656157 anti-tumor activity, it also significantly inhibited pancreatic function, as well as after GSK2656157 withdrawal the anti-tumor activity was reversed [284]. Hence there is a need to conduct further research in order to find an alternate to GSK inhibitors that is not detrimental to normal cells.

On the other hand, it has recently been reported that there is a connection between the UPR signaling pathway and the tumor necrosis factor (TNF), implicated both in inflammation and cell death. Research by Rojas-Rivera et al. [286] has demonstrated that both GSK2606414 and GSK2656157, commonly used as inhibitors of the PERK-dependent UPR signaling pathway, trigger complete inhibition of the TNF-mediated receptor-interacting serine/threonine-protein kinase 1 (RIPK1)-dependent cell death in the nanomolar range and the above-mentioned inhibitory effect was independent of PERK inactivation. Moreover, GSK2606414 and GSK2656157 inhibited TNF-mediated RIPK1-dependent cell death at a concentration which did not affect PERK activity within cells. Thereby, it may be concluded that GSK2606414 and GSK2656157 constitute direct small-molecule inhibitors of RIPK1 with similar potency to the already existing RIPK1 inhibitor, GSK′963. These findings have important consequences due to the risk of misinterpretation while using GSK2606414 and GSK2656157 as selective inhibitors of PERK. In addition, only AMG’44 is characterized by high inhibitory activity against PERK without interfering with RIPK1, hence it is recommended to use AMG′44 for selective PERK inhibition rather than GSK inhibitors [286].

Recently, the novel inhibitors of the ATF6 pathway were also identified, but their mechanism of action has not yet been determined [287]. 

Moreover, it has been reported that evodiamine (EVO), derived from the Evodia rutaecarpa plant, possesses an ability to induce apoptosis in human cancer cells, especially ovarian (the study involved A2780, A2780CP, ES-2, and SKOV-3 cell lines) and renal carcinoma (RCC) (A498, 786-O, ACHN, and Caki-1) [281,288]. In CRC cells, JNK/PERK-mediated phosphorylation of the BCL-2 protein participated in EVO-induced apoptosis and G2/M arrest involving tubulin polymerization. Interestingly, it has been found that EVO activates both JNK and PERK accordingly by increasing their phosphorylation. Then, phosphorylation of BCL-2 (Ser-70) occurs, resulting in disruption of the mitochondrial membrane potential (MMP) and eventually in apoptosis. There is strong evidence that JNK has a pro-apoptotic role in various cells, since mice with knocked-out JNK genes (JNK^−/−^) proved to be resistant to apoptosis induced by chemicals and irradiation. However, numerous studies should be undertaken to explain the underlying mechanism of the above-mentioned event. The EVO, EVO-7, and EVO-8-dependent increased expression of p-PERK at Thr-980, p-JNK, and cleavage of poly (ADP-ribose) polymerase (PARP) could be suppressed by addition of the PERK inhibitor GSK2606414. We can therefore conclude that PERK indeed stimulates, directly or indirectly, JNK and Bcl-2 protein phosphorylation [281].

Aloe-emodin (1, 3, 8-trihydroxyanthraquinone, AE), naturally extracted from aloe vera, was discovered to suppress proliferation of tumor cells and accelerate apoptosis. AE-induced ROS generation have been so far demonstrated in human lung squamous cells, human OS cells, as well as in two CRC cell lines, SW620 and HT29. It has been reported that exposure of cells to AE triggers intracellular ROS generation and subsequently activates an ER stress-dependent apoptotic cell death, which was confirmed by enhanced expression of the ER stress-related proteins, including GRP78, p-PERK, p-eIF2α, and CHOP. Moreover, not only increased cytosolic calcium content was observed, followed by the upregulation of the calpain-1, calpain-2 and caspase-12, but also an intracellular hydrogen peroxide and superoxide accumulation [86].

Curcumin-derived compound, WZ35, exhibited significantly better ER stress-dependent apoptotic effect than curcumin in human prostate cells [289]. It has also been reported to have anti-neoplastic activity against following CRC cell lines: HCT116, SW620, and CT26. Nevertheless, since little is known about its impact on the other cell lines, it is worthy of further investigation. Similar to AE, WZ35 treatment induced apoptosis via ROS-ER stress-mediated mechanism, in CT26 colon cancer (both in in vitro and in vivo models), as well as in the gastric cancer cells. WZ35-induced cell cycle arrest in G2/M phase was also observed and it was strictly correlated with a significant suppression of the mouse double minute 2 homolog (MDM-2), Cyclin B1 and Cyclin-dependent kinase 1 (Cdc2) expression as well as increased activation of caspase-3 and PARP. Interestingly, as in the case of EVO, the treatment effect was reversible via inhibited CHOP expression preceded by the inhibition of p-PERK by GSK2606414, which may suggest a dual role of PERK in development of targeted anti-cancer therapy. In spite of that, study claims that ER stress might not be the only ROS-mediated pathway of WZ35 effect with its accurate ROS-producing mechanism remaining unclear [267].

On the other hand, aforesaid Gen, a phytoestrogen extracted from soy bean, has been proven to affect the expression of estrogen receptors and some of tumor suppressor genes in hormone-dependent tumor types [290,291]. It also decreases production of ROS via regulating antioxidant enzymes activity, and hence suppresses the tumor growth [292,293]. However, new findings suggest that Gen also exhibits pro-oxidant properties and may induce ROS-mediated apoptosis, depending on the concentration used and the cell type [294,295,296,297]. At the molecular level, it was also proven that Gen targets multiple cellular signaling pathways correlated with cell cycle and apoptosis, such as caspases, Bcl-2 family members, NF-κB, PI3K/Akt or mitogen-activated protein kinase (MAPK) [298]. A study by Hsiao et al. [299] has demonstrated that Gen induced G2/M cell cycle arrest and evoked apoptosis in human leukemia HL-60 cells by an ER stress- and mitochondria-dependent signaling pathway, which was confirmed by the increased expression of IRE1α, calpain-1, GRP78 and ATF6, as well as apoptosis-related proteins [299].

## 7. Summary and Perspective

Since UPR is capable of triggering both pro-survival and pro-apoptotic signals, it is still unclear whether the protective elements of the response are enhanced or the destructive ones suppressed in order to prevent the UPR-induced apoptosis [132]. To determine cell fate, each factor may transcriptionally activate a certain target in its own unique pathway, as well as demonstrate diverse mechanisms of action towards other targets [27]. Conversely, many downstream target genes are regulated individually by numerous pathways, some of which even require simultaneous activation of more than one signaling pathway. This is also why altered signaling through the other branches frequently emerges to be an outcome of one-branch inhibition.

Therefore, there is an obvious link among all three UPR signaling branches. CHOP, for instance, serves as a downstream target of PERK, IRE1α, and ATF6, for which reason silencing a single UPR effector is insufficient to suppress ER stress-induced apoptosis [216,300]. The major problem in studies conducted hitherto is that only the expression of two to three ER stress markers was investigated and still more data is required to be gathered. Moreover, commonly used in studies inducers of ER stress, including thapsigargin and tunicamycin, were revealed to complicate the research due to their pleiotropic effects and acute toxicity [7,301]. In future therapy, it is highly recommended to develop a drug which selectively targets a single branch of the UPR signaling pathway. PERK seems to be a favorable candidate for that purpose in the context of small-molecule inhibitors, since IRE1α and ATF6 present remarkably weaker activities during chronic ER stress [300]. Such selective UPR inhibition would be a promising therapeutic target to promote apoptosis, decrease cellular pro-survival mechanisms, or cause the UPR overload. However, the current problem is that certain UPR downstream effectors have been confirmed to both prevent ER stress-mediated apoptosis and promote tumor growth unintentionally, in some circumstances. PERK-based therapies, for instance, are expected to drive tumor cells into dormancy, thereby inducing chemotherapy resistance [302]. It is equally important that UPR modulators ought to be highly selective against tumor tissue, considering that there are several normal cell types (e.g., B lymphocytes or pancreatic β-cells) possessing high requirement of the ER to fold proteins, thus the prospective therapy would necessarily need close monitoring [303]. In order to monitor temporal dynamic, time-dependent effects of future drugs and potential toxicities, the use of experimental models is required.

Taken together, the UPR signaling pathway represents a promising potential cancer therapeutic strategy, although particular vigilance should be exercised in respect of its opposing effects on either neoplastic or normal cells proliferation and survival. With a view to the lack of both in vivo and branch-specific research, as well as the temporal UPR dynamics in cancer remaining unknown, more profound studies need to be done in order to elucidate the interplay between UPR and other pathways. Further research should be conducted to gather detailed knowledge of how tumor cells adapt to long-term ER stress, determine where the survival/apoptosis balance lies and whether the reinforcement of protein-folding capacity would prevent cancer incidents during carcinogen exposure.
